# Male germ cell proliferation and apoptosis in sexually immature meagre *Argyrosomus regius* (Asso, 1801) treated with recombinant follicle stimulating hormone

**DOI:** 10.1038/s41598-023-34102-1

**Published:** 2023-04-28

**Authors:** Rosa Zupa, Neil Duncan, Ignacio Giménez, Constantinos C. Mylonas, Chrysovalentinos Pousis, Letizia Passantino, Rezart Cuko, Aldo Corriero

**Affiliations:** 1 Department of Veterinary Medicine, University of Bari Aldo Moro, S.P. per Casamassima km.3, 70010 Valenzano, Bari Italy; 2 IRTA, Ctra. de Poble Nou km. 5.5, 43540 La Ràpita, Tarragona Spain; 3 Rara Avis Biotec, S. L., Calle Moratín 17, 46002 Valencia, Spain; 4 Institute of Marine Biology, Biotechnology and Aquaculture, Hellenic Centre for Marine Research, 71003 Heraklion, Crete Greece; 5 DiMePRe-J, University of Bari Aldo Moro, S.P. per Casamassima km.3, 70010 Valenzano, Bari Italy

**Keywords:** Microscopy, Histology, Animal biotechnology

## Abstract

The meagre *Argyrosomus regius* (Asso, 1801) is a marine fish species that has an increasing aquaculture production in Europe. Lowering the age at maturity of hatchery-produced juveniles would support meagre aquaculture by reducing time between generations in selective breeding programs and reducing industrial costs for broodstock maintenance. The aim of this work was to assess the effects of a treatment with recombinant follicle stimulating hormone (rFsh), produced in ovarian cells of Chinese hamsters, on male germ cell proliferation and apoptosis in sexually immature meagre. The rFsh-treated fish had higher gonadosomatic index, larger seminiferous tubules, more abundant luminal spermatozoa, a lower density of anti-PCNA positive single A spermatogonia, a higher density of anti-PCNA positive spermatocysts and a lower incidence of germ cell apoptosis than control groups. The present study demonstrated the effectiveness of the produced rFsh in stimulating testis development and spermatogenesis in pre-pubertal meagre. Moreover, the rFsh treatment proved to be highly efficient in removing the apoptotic block of spermatogenesis observed in juvenile meagre, allowing spermatogonial survival and progress towards meiosis. The administration of rFsh did not stimulate spermatogonial self-renewal, a process whose control still needs to be elucidated.

## Introduction

The meagre *Argyrosomus regius* (Asso, 1801) is distributed in the Mediterranean Sea and in the eastern Atlantic Ocean from Norway to Congo. A member of the Sciaenidae family, it is generally found in coastal marine waters at all depths of the water column including brackish estuaries and lagoons^1^. Meagre has high-quality flesh that consumers appreciate, and meagre produced from aquaculture supply lucrative European niche markets with increasing retail prices that in 2022 reached 20 euro/kg (author’s personal observation in Italian fish shops and supermarkets). In 2020, European meagre aquaculture production increased to about 9200 tonnes that was mainly concentrated in Spain and Greece (85% of the European production) followed by France, Croatia, Italy and Portugal^2^. On the other hand, meagre production in Egypt has reached 26,000 tonnes in the same year^3^.

Meagre reproduction in captivity progresses without problems through to the final stages of vitellogenesis and spermatogenesis, but then hormonal treatments are frequently required to induce oocyte maturation, ovulation and spawning^4–12^. Although occasional spontaneous spawning has been reported in captivity^8^, females commonly exhibit arrested maturation close to the initiation of oocyte maturation^5–12^ and male sperm production has been low^10^. These captivity related reproductive dysfunctions have been overcome with the administration of a single injection of 15 µg kg^−1^ of gonadotropin releasing hormone agonists (GnRHa)^6,10,11^. The hormone treatments have proved very successful, and individual females have been induced to spawn each week for up to 17 weeks without any detrition in egg quality^10^, and protocols have been adapted to provide families for breeding programs by pairing breeders in individual tanks^6^ or using in vitro fertilisation^12^. Using fertilised eggs produced through hormonal induction of spawning, larval rearing protocols have been set up in many Mediterranean countries, including locations in the Canary Islands^5,13^.

In vertebrates, the attainment of puberty, i.e. the physiological changes through which individuals become sexually mature, involves the activation of the brain-pituitary-gonad axis, a process characterised by the release of pituitary gonadotropins (Gths; follicle stimulating hormone, Fsh and luteinizing hormone, Lh). Gonadotropin release, controlled by the hypothalamus through GnRH, in turn induces gonad development and gametogenesis through the stimulation of sex steroid hormone synthesis^14–16^. Meagre sexual maturity is attained at 3–4 years of age^5,17^, which means that breeding selection programs have a rather long generation time. Lowering the age at maturity of hatchery-produced juveniles would support meagre aquaculture by reducing generation time in selective breeding programs.

Over the years, the induction of precocious puberty in fishes has been attempted through the use of GnRHa, gonadotropins or steroid hormones. While treatment with GnRHa did not succeed in inducing oogenesis in red sea bream *Pagrus major*^18^ or striped bass *Morone saxatilis*^19^, while treatments with Fsh have been more effective in inducing precocious gonad development in fishes^20–23^, although with severe limitations due to the species-specificity of gonadotropin structure. Treatments with testosterone (T) showed negligible effects on gametogenesis of juvenile striped bass^24^ and, in general, steroid hormone administration induces downregulation of different genes involved in the reproductive function^25–27^.

More recently, the use of recombinant Gths (rGths), has been successfully used to ameliorate reproductive dysfunctions in adult cultured fish and recombinant Fsh (rFsh) has been used to stimulate spermatogenesis in Senegalese sole *Solea senegalensis*^28,29^, European eel *Anguilla anguilla*^30^ and flathead grey mullet *Mugil cephalus*^31,32^. The aim of this work was to assess the effects that a 6-week rFsh treatment had on germ cell proliferation and apoptosis in sexually immature meagre in an effort to set up a protocol for the induction of precocious puberty.

## Methods

### Ethics

The study was in accordance with the European Directive, the Spanish Royal Decree and the Catalan Law for the protection of animals used for scientific purposes. The present study was approved by IRTA’s (Institute of Agrifood Research and Technology) Committee of Ethics and Experimental Animal (CEEA) and the Catalan Government as experimental project 11,264 with expedient number FUE-2020-01,809,522 and ID CJQX0B0PH. The authors complied with the ARRIVE guidelines.

### Recombinant Fsh production

Fsh α and β subunits amino acid sequences were deduced from the meagre pituitary mRNA sequences deposited in the European Nucleotide Archive (ENA) under the project accession number PRJEB57583.

The deduced sequences were:

Glycoprotein hormone α mature subunit:

- YPNIELSNMGCEECTLRKNSVFSRDRPVYQCMGCCFSRAYPTPLKAMKTMTIPKNITSEATCCVAKHSYEIEVAGIRVRNHTDCHCSTCYFHKI

Fsh β subunit:

- MQLVVMAAVLAVAGAWQGCGFDCHPTNISIPVESCGNTEFIETTICAGQCYHEDPVYIGHDDWVEQRTCNGDWSYEVKHIKGCPVGVTYPVARNCKCTACNAGSTYCGRFPGDVSSCLSF

Single-chain meagre rFsh was produced by Rara Avis Biotec S.L (Valencia, Spain) as described for the flatfish *Senegalese sole*^28^. In brief, Chinese hamster ovary (CHO-S) cells in suspension were transfected with an expression construct encoding a fusion protein containing the entire coding sequence of meagre Fshβ subunit, the 28 amino acids of the carboxyl-terminal sequence of the human chorionic gonadotropin (hCGβ) β subunit as a linker, and the mature sequence of meagre Fshα subunit (Cga). After 120 h of CHO cell culture, ion exchange chromatography was used to purify the secreted recombinant hormones from the culture medium. The hormones were concentrated to 12 µg mL^−1^ and quantified by semiquantitative Western blot, using polyclonal (mouse) antibody against meagre Fshβ subunit and metal affinity purified His-tagged meagre rFsh as standard.

### Fish rearing and rFsh administration

During November 2020, the fish were transported to IRTA (Institute of Agrifood Research and Technology), La Ràpita, Spain, from the Portuguese Institute for Sea and Atmosphere (IPMA), Olhão, Portugal. The fish were hatched in the spring 2020 and reared in IPMA to approximately 200 g. In IRTA all fish were tagged with a Passive Integrated Transponder (PIT) tag (Trovan^®^, ZEUS Euroinversiones S.L. Madrid, Spain) for identification. Prior to the experiment the fish were grown from approx. 200 g to 1.07 ± 0.18 kg and 43.4 ± 2.7 cm (27 October 2021) under natural conditions (photoperiod and temperature), in the same 10-m^3^ tank (rectangular 1 × 2 × 5m) that was used for the experiment. The tank was connected to a recirculation system (IRTAmar^®^) to control water quality and temperature. During the experiment the temperature was maintained at 18.1 ± 0.3 °C and the photoperiod was natural. Fish were fed 2 times a day, 6 days a week to satiation with a pelleted diet Brood Feed Lean (SPAROS, Portugal). The fish were starved for 24 h before experimental sampling. Fish were anaesthetised with 70 mg L^−1^ of MS-222 for all sampling procedures and hormone injection. To obtain tissues, fish were sacrificed with an overdose of anaesthesia followed by pithing to destroy the brain.

### Experimental design and sampling

The experiment had a duration of 6 weeks during which a group of meagre were treated with rFsh and a control group was treated with saline solution. The rFsh was administered weekly initially with increasing doses (week 1: 6 µg kg^−1^ and week 2: 9 µg kg^−1^) followed by a constant dose of 12 µg kg^−1^ (weeks 3–6). The control fish were given a weekly 1 mL injection of saline solution. The experimental groups were sampled on two occasions at the start of the experiment (T = 0) and after 6 weekly injections. On 27 October 2021, untreated control fish (“CONTROL 0” group) were sampled in order to assess the baseline reproductive state on the same day the treated group received the first rFsh administration and the control group the first saline solution injection. Six weeks after the first rFsh administration, on 10 December 2021, control fish (“CONTROL 6” group) and rFsh-treated fish (“TREATED” group) were sampled to determine the effect of the rFsh treatment. A total of 12 fish were randomly selected for each sample point and once sacrificed the fish were sexed by macroscopic and microscopic observations of the gonads. Male fish were selected from the 12 sacrificed fish and only male fish were used in the present study. Eighteen juvenile meagre males (18 months of age) were sampled, for the present study. At the start of the experiment, group CONTROL 0, six males were obtained; however, one CONTROL 0 male fish was removed from the experiment (i.e. n = 5) because it had abnormally high testis mass and gonadosomatic index, and it was considered as an individual undergoing precocious puberty. On week six, nine male control fish were obtained from CONTROL 6 and four rFsh treated male fish from TREATED group, respectively. From each fish, total length (TL, in cm), body mass (BM, in g) and gonad mass (GM, in g) were measured (Table 1) and gonadosomatic index (GSI) was calculated as 100 × GM/BM. From each fish, 1-cm thick gonad slices were cut, fixed in Bouin's solution, and destined to histological analysis.

**Table 1:** Biometric data of control and rFsh-treated juvenile meagre males.

Group	Sampling date	Total length (cm)	Body mass (g)	Gonad mass (g)
Control 0 (N = 5)	27/10/2021	48	1374	0.8
		42	1073	3.6
		38	690	3.3
		44	1090	1.1
		46	1193	0.9
Mean (± sd)		43.6 ± 3.8	1084.0 ± 250.7	1.9 ± 1.4
Control 6 (N = 9)	10/12/2021	56	1577	2.9
		51	1239	1.0
		44	869	1.5
		50	1173	1.2
		54	1435	1.0
		49	1105	1.4
		48	909	0.5
		49	1061	1.3
		43	758	2.7
Mean (± sd)		49.3 ± 4.2	1125.1 ± 266.2	1.5 ± 0.8
Treated (N = 4)	10/12/2021	50	1135	6.5
		44	772	4.8
		43	776	10.8
		47	944	8.7
Mean (± sd)		46.0 ± 3.2	906.8 ± 172.0	7.7 ± 2.6

### Histology, immunohistochemistry and identification of apoptotic germ cells

Testis samples were dehydrated in ethanol, clarified in xylene and embedded in paraffin wax. Four-μm thick sections were stained with haematoxylin–eosin (H–E) or processed for immunohistochemistry and for the identification of apoptotic germ cells.

The identification of proliferating germ cells was performed through the immunohistochemical localization of the Proliferating Cell Nuclear Antigen (PCNA), a polymerase delta accessory protein used as marker of proliferation, according to the procedure described in Zupa et al.^33,34^. Briefly, sections were pre-treated for 10 min with 3% H_2_O_2_ to inhibit endogenous peroxidase, incubated for 30 min in normal horse serum (NHS; Vector, Burlingame, Ca) and then incubated overnight in a moist chamber at 4 °C with monoclonal antibodies to PCNA (Santa Cruz Biotechnology Inc., Dallas, Texas) diluted 1:25 in phosphate buffered saline (PBS; 0.01 M, pH 7.4, containing 0.15 M NaCl) containing 0.1% bovine serum albumin (BSA; Sigma-Aldrich, Milan, Italy). The immunohistochemical reaction was visualized through the avidin–biotin-peroxidase complex (ABC) procedure using the Vectastain Universal Elite Kit (Vector, Burlingame, Ca). Peroxidase activity was visualized by incubating for 10 min with a 3,3′-diaminobenzidine (DAB) Peroxidase Substrate Kit (Vector, Burlingame, Ca). To confirm the specificity of the immunoreaction, control procedures were carried out by replacing the primary antibody with NHS and PBS.

Apoptotic germ cells were identified through the terminal deoxynucleotidyl transferase-mediated d’UTP nick end labeling (TUNEL) method with an in situ Cell Death Detection Kit, AP (Roche Diagnostics, Mannheim, Germany)^33,34^. Prior to incubation with the reaction mixture, the sections were incubated in a permeabilization solution of 0.1% Triton X-100 in 0.1% sodium citrate for eight min. Terminal deoxynucleotidyl transferase was diluted 1:2 in TUNEL Dilution Buffer (Roche Diagnostics, Mannheim, Germany). A ready-to- use solution of nitro-blue tetrazolium chloride/5-bromo-4-chloro-3′-indolyphosphate p-toluidine salt (NBT/BCIP) (Roche Diagnostics, Mannheim, Germany) served as a substrate for the signal conversion.

### Seminiferous tubule diameter and quantification of germ cell proliferation and apoptosis

Seminiferous tubule diameter was measured, and proliferating and apoptotic germ cells were quantified according to Zupa et al.^33,34^. The diameter of at least 80 seminiferous tubules per testis was measured from the sections used for germ cell proliferation and apoptosis analyses. The density of anti-PCNA positive single type A spermatogonia (number of cells/mm^2^ germinal epithelium) and the density of anti-PCNA positive spermatocysts (i.e. number of spermatocysts containing spermatogonia or primary spermatocytes/mm^2^ germinal epithelium), as well as the surface occupied by TUNEL positive cells (μm^2^/mm^2^ germinal epithelium), were measured on 5–10 randomly selected fields of each testis section.

All the above measurements were carried out from microphotographs taken with a digital camera (DFC 420; Leica, Cambridge, UK) connected to a light microscope (DIAPLAN; Leitz, Wetzlar, Germany), using the interactive measurement function of an image analysis software (Leica Application Suite, version 3.3.0; Cambridge, UK).

### Statistical analysis

Differences in GSI, seminiferous tubule diameter, density of anti-PCNA positive single A spermatogonia, density of anti-PCNA positive spermatocysts and surface occupied by apoptotic germ cells between groups were assessed by an ANOVA followed by Duncan’s new multiple range post hoc test. Prior to the ANOVA, normality of variance was assessed through Shapiro–Wilk W test and GSI and apoptosis data were arcsine transformed, as appropriate with proportions^35^. All the results are presented as means ± sd, and the statistical probability significance was established at the *P* < 0.05 level. Caution is required when interpreting comparisons that were not significantly different amongst the density of anti-PCNA positive spermatocysts because the power of the ANOVA test was 0.73. The power of all the other ANOVA tests was > 0.8.

## Results

### Gonadosomatic Index, testis histology and diameter of seminiferous tubules

**Figure 1:**
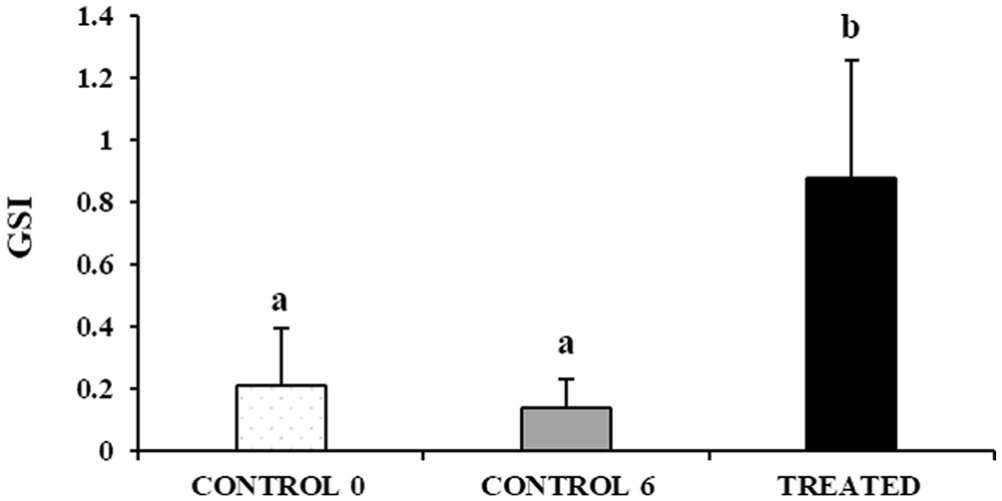
Mean (± sd) gonadosomatic index (GSI) of untreated (CONTROL 0, n = 5), treated with saline solution for 6 weeks (CONTROL 6, n = 9) or treated with rFsh for 6 weeks (TREATED, n = 4) juvenile meagre males. Different letters represent statistically significant differences (ANOVA; *P* < 0.05).

Gonadosomatic index did not change significantly (*P* = 0.37) between the CONTROL 0 (0.21 ± 0.19) and CONTROL 6 (0.14 ± 0.09) groups. On the other hand, the TREATED fish had significantly higher GSI than both CONTROL groups (0.88 ± 0.38, *P* < 0.05) (Fig. 1).

**Figure 2:**
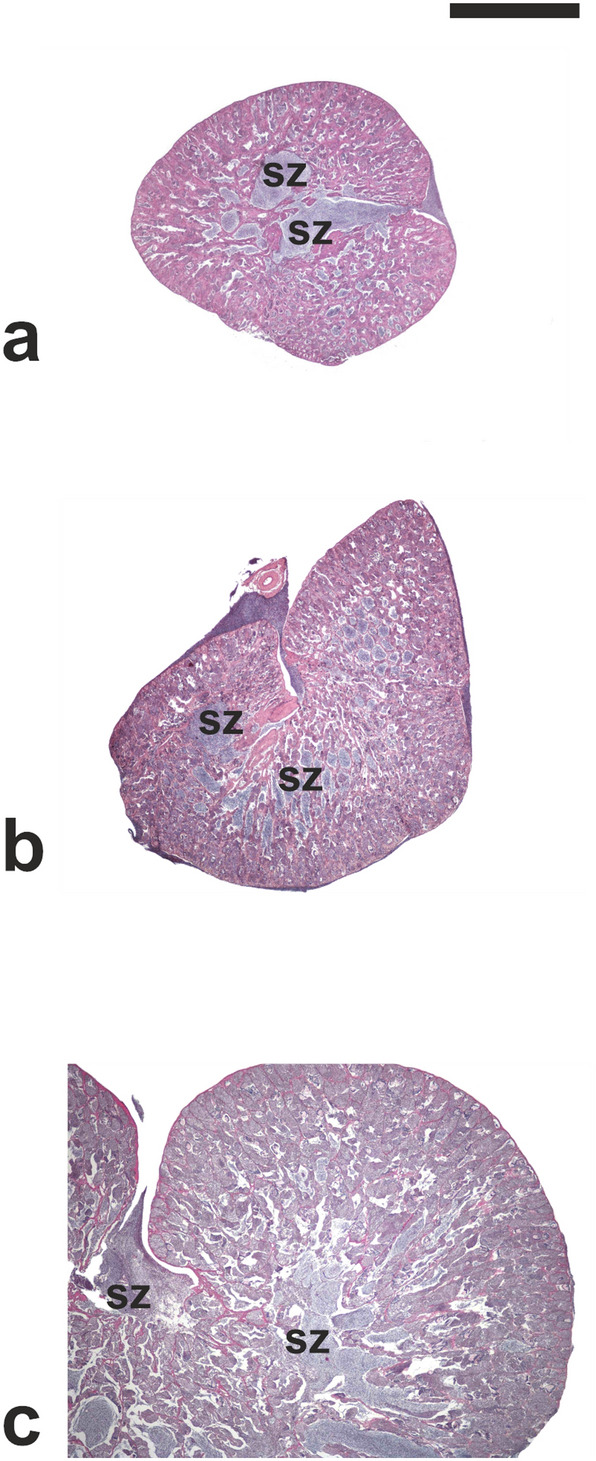
Micrographs of testis sections from juvenile meagre. (**a**) Testis section of an untreated fish (CONTROL 0 group); (**b**) Testis section of a fish treated with saline solution for 6 weeks (CONTROL 6 group); (**c**) Testis section of a fish treated with rFsh for 6 weeks (TREATED group). H-E staining. Magnification bar = 1 mm. sz = spermatozoa.

**Figure 3:**
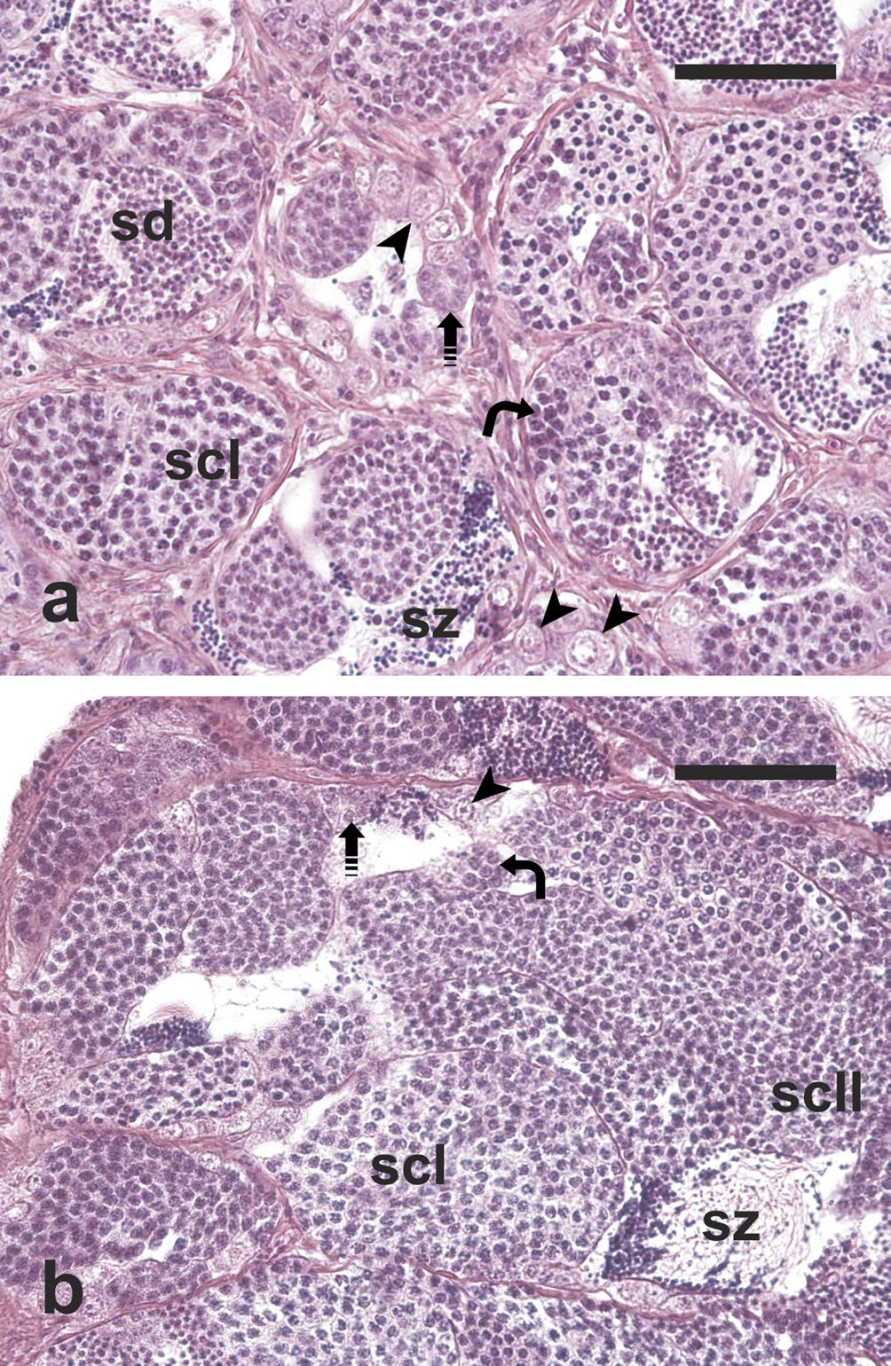
Micrographs of testis sections from (**a**) a juvenile meagre treated with saline solution for 6 weeks (CONTROL 6 group) and (**b**) a juvenile fish treated with rFsh for 6 weeks (TREATED group). H–E staining. Magnification bars = 50 μm. Arrowhead = single type A spermatogonium; dashed arrow = type A spermatogonial cyst; curved arrow = type B spermatogonial cyst; scI = primary spermatocyte cyst; scII = secondary spermatocyte cyst; sd = spermatid cyst; sz = spermatozoa.

**Figure 4:**
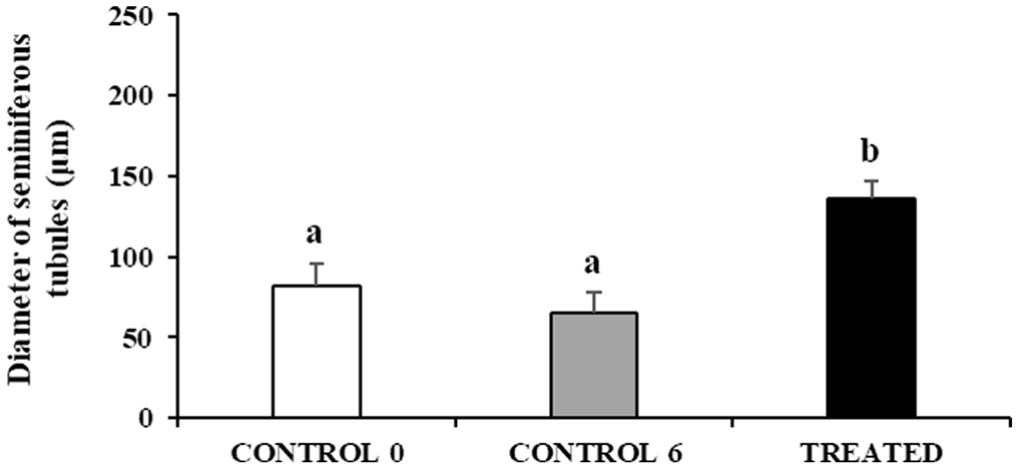
Mean (± sd) diameter of seminiferous tubules of untreated (CONTROL 0, n = 5), treated with saline solution for 6 weeks (CONTROL 6, n = 9) or treated with rFsh for 6 weeks (TREATED, n = 4) juvenile meagre males. Different letters represent statistically significant differences (ANOVA; P < 0.05).

Fish from CONTROL 0 and CONTROL 6 groups had small testes containing germ cells in all spermatogenic stage, including limited numbers of spermatozoa (Fig. 2a, b). Although small numbers of spermatozoa could be observed in the sperm duct system (Fig. 2a,b), most of seminiferous tubules showed no lumen or a very small lumen (Fig. 3a). Fish belonging to the TREATED group showed larger testes compared to the two CONTROL groups (Fig. 2c) and more active spermatogenesis characterised by a thicker germinal epithelium and a higher number of large spermatocyte, spermatid and sperm cysts, as well as more abundant luminal spermatozoa (Fig. 3b). The diameter of seminiferous tubules was similar in the two CONTROL groups (81.6 ± 14.4 vs 64.9 ± 13.6 μm; *P* = 0.05). Seminiferous tubules were significantly larger in TREATED fish compared with both controls (136.1 ± 10.8 μm, *P* < 0.05) (Fig. 4).

### Germ cell proliferation and apoptosis

**Figure 5:**
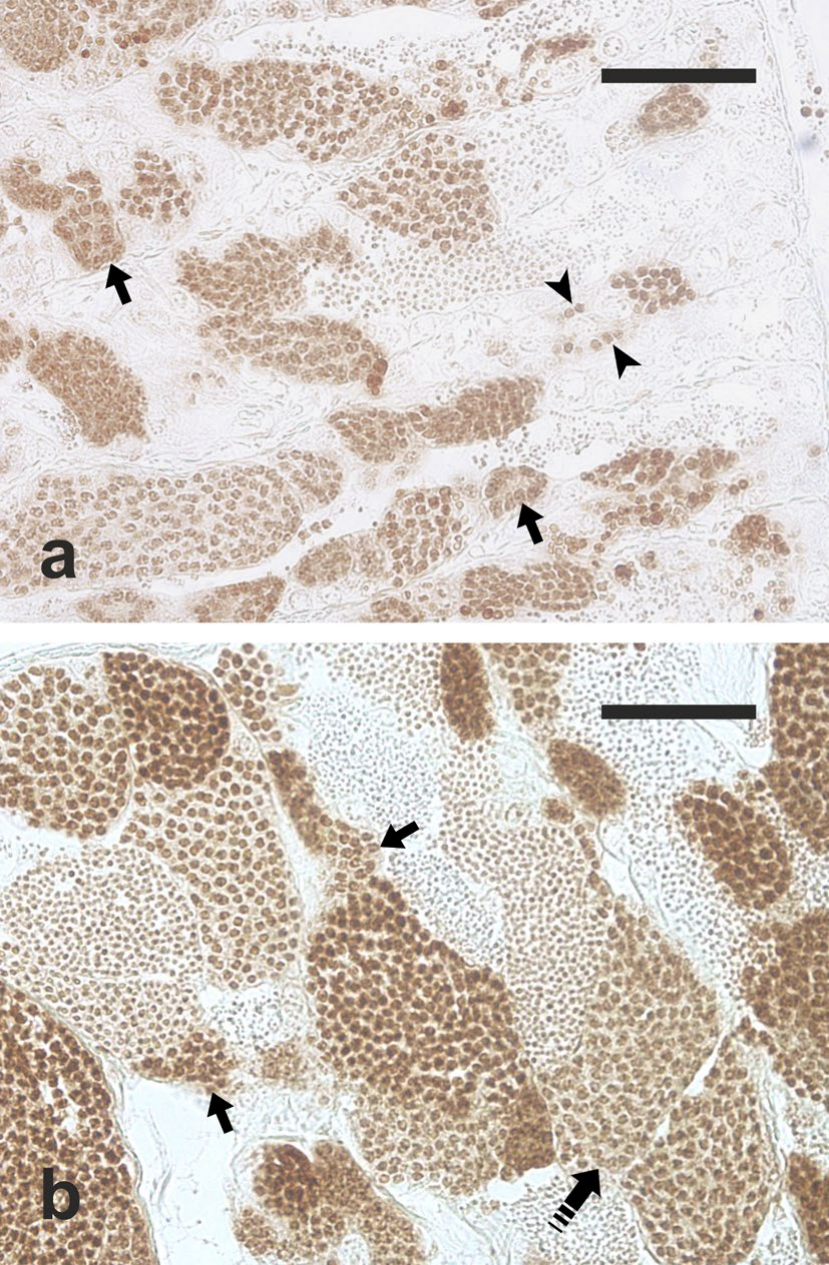
Micrographs of testis sections of (**a**) a juvenile meagre treated with saline solution for 6 weeks (CONTROL 6 group) and (**b**) a juvenile fish treated with rFsh for 6 weeks (TREATED group), immunostained with antibodies against the proliferating cell nuclear antigen (PCNA), which stains brown the nuclei of proliferating cells. Magnification bars = 50 μm. Arrowhead = anti-PCNA–positive single type A spermatogonium; arrow = anti-PCNA–positive spermatogonial cyst; dashed arrow = anti-PCNA–positive primary spermatocyte cyst.

Anti-PCNA immunostaining was observed in the nuclei of single type A spermatogonia, and spermatogonia contained in cysts and in primary spermatocytes (Fig. 5a,b). A weak staining of the nuclei of secondary spermatocytes was also observed, but these cells were not included in the quantitative analysis.

**Figure 6:**
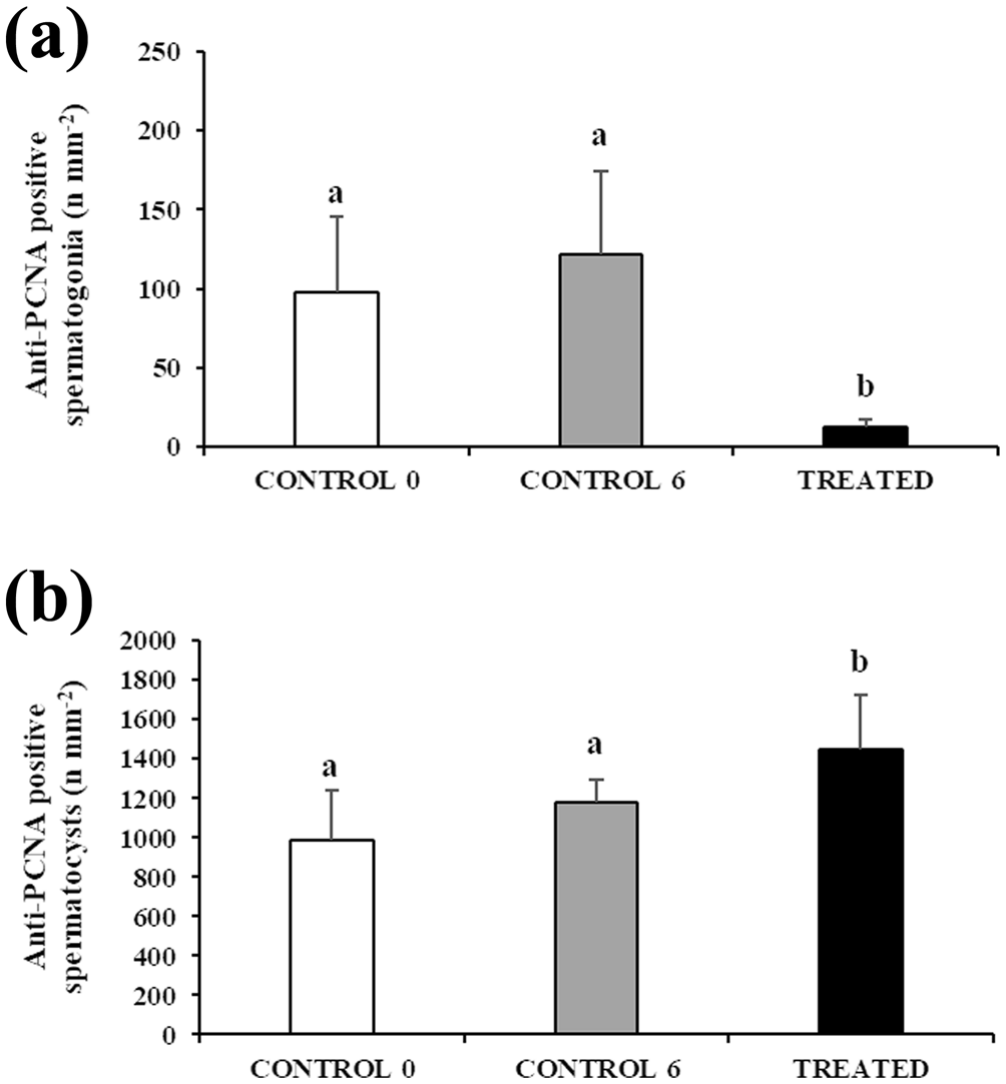
Changes in mean (± sd) anti-proliferating cell nuclear antigen (PCNA) positive germ cell density in untreated (CONTROL 0, n = 5), treated with saline solution for 6 weeks (CONTROL 6, n = 9) or treated with rFsh for 6 weeks (TREATED, n = 4) juvenile meagre males. (**a**) Anti-PCNA positive single A spermatogonia. (**b**) Anti-PCNA positive spermatocysts. Different letters represent statistically significant differences (ANOVA; *P* < 0.05).

A relative quantification of anti-PCNA positive single type A spermatogonia and spermatocysts (cysts containing spermatogonia or primary spermatocytes) in the three experimental groups was undertaken (Fig. 6; Supplementary File S1). No statistically significant difference was observed in the density of anti-PCNA positive single type A spermatogonia and anti-PCNA positive spermatocysts between the two CONTROL groups (98.2 ± 47.2 vs 121.3 ± 52.9 n/mm^2^, *P* = 0.38 and 988.8 ± 251.5 vs 1179.1 ± 113.9 n/mm^2^, *P* = 0.11, respectively). The TREATED group had a significantly lower density of anti-PCNA positive single type A spermatogonia (12.4 ± 4.8 n/mm^2^) (Fig. 6a) and a significantly higher density of anti-PCNA positive spermatocysts (1449.5 ± 279.2 n/mm^2^) (Fig. 6b) compared with both CONTROL groups (*P* < 0.05 in all comparisons).

**Figure 7:**
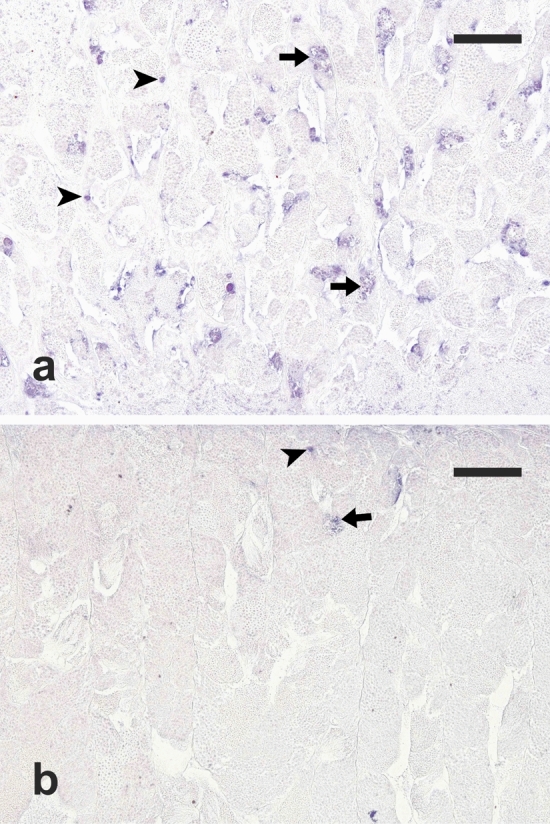
Micrographs of testis sections from (**a**) a juvenile meagre treated with saline solution for 6 weeks (CONTROL 6 group) and (**b**) a juvenile meagre treated with rFsh for 6 weeks (TREATED group), stained with the terminal deoxynucleotidyl transferase-mediated 2′-deoxyuridine 5′-triphosphate nick end labeling (TUNEL) method, with apoptotic cells appearing as dark blue dots. Magnification bars = 100 μm. Arrow = TUNEL–positive spermatogonial cyst; arrowhead = TUNEL–positive single type A spermatogonium.

**Figure 8:**
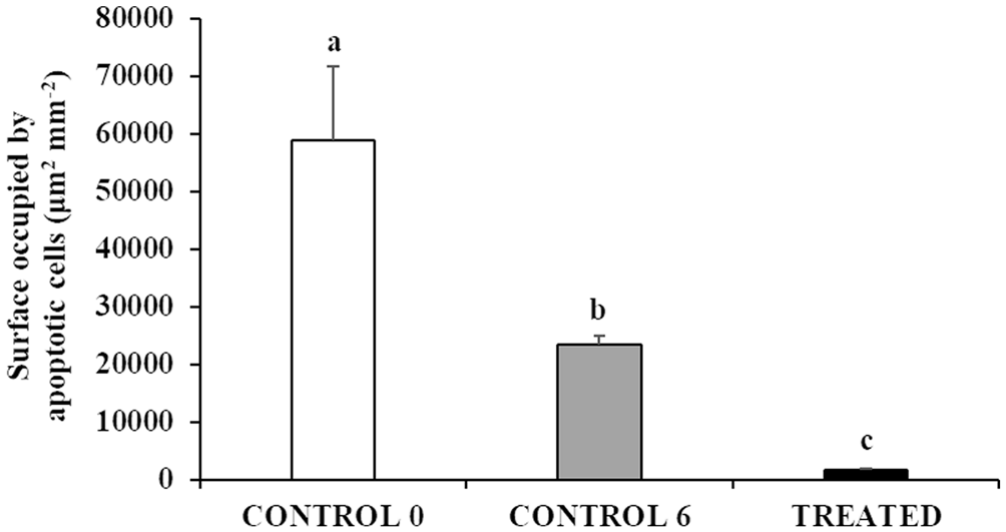
Changes in mean (± sd) surface occupied by apoptotic germ cells in untreated (CONTROL 0, n = 5), treated with saline solution for 6 weeks (CONTROL 6, n = 9) and treated with rFsh for 6 weeks (TREATED, n = 4) juvenile meagre males. Different letters represent statistically significant differences (ANOVA; *P* < 0.05).

All the analysed testes showed apoptotic germ cells in the tubular compartment. The TUNEL reaction involved mainly single A spermatogonia and spermatogonia contained in cysts. When apoptosis involved a spermatocyst, all the spermatogonia contained in the cysts were apoptotic (Fig. 7). A high level of apoptosis was observed in the CONTROL groups and apoptotic cells were observed in most of the seminiferous tubules (Fig. 7a) whereas TUNEL-positive germ cells were rarely observed in the TREATED group (Fig. 7b). In fact, the semi-quantitative analysis of apoptotic cell density showed that the surface occupied by apoptotic germ cells was significantly lower in the TREATED group (1700.8 ± 297.3 μm^2^/mm^2^) compared with both CONTROL 0 (58,799.8 ± 12,903.7 μm^2^/mm^2^) and CONTROL 6 (23,451.4 ± 1554.8 μm^2^/mm^2^) (*P* < 0.05 for both comparisons) (Fig. 8).

## Discussion

In recent years, in vivo experiments with sexually immature male fish have demonstrated the effectiveness of rFsh produced with different biotechnologies in inducing testicular growth and the onset of spermatogenesis^29–32,36–38^. In the present study, a statistically significant GSI increase was observed in 18-month-old pre-pubertal meagre males treated for 6 weeks with rFsh produced in a CHO-S cell line, confirming the effectiveness of the rFsh treatment in triggering the onset of testis growth and spermatogenesis. The histological analysis of the testes showed that rFsh-treated fish had a more active spermatogenesis compared to the control males, with larger seminiferous tubules containing abundant luminal spermatozoa. However, the GSI of rFsh-treated fish was lower than adult fish in advanced reproductive maturation^39^. The lower GSI can be attributed to the short 6-week rFsh treatment, since complete testes development in adult males takes place in response to elevated 11-ketotestosterone (11-KT) for 2–3 months^39^. In addition, low GSI (0.2–0.7) has been observed in pubertal males during the first year of maturation^39^ and GSI ≈ 2.0 was found in fish from the same cohort of this experiment (N.D. unpublished data). However, the present study has, in agreement with other studies^28–32^, demonstrated that a 6-week rFsh treatment stimulated testicular development, towards an advanced stage of spermatogenesis.

In fish testes, one can find different types of spermatogonia^40^, such as single undifferentiated type A spermatogonia (stem spermatogonia; SpA_und_), differentiated type A spermatogonia (SpA_diff_) (germ cells already committed towards spermatogenesis), and type B spermatogonia. Single undifferentiated type A spermatogonia are germ cells residing in a specific microenvironment created by a supporting Sertoli cell, called ‘niche’^40^; SpA_diff_ are found in cysts with two to eight cells and type B spermatogonia occur in cysts with 16 or more cells, depending on the species-specific number of mitotic cell cycles. The present data clearly indicate that treatment with rFsh was highly effective in stimulating (a) transition from SpA_und_ to SpA_diff_, (b) proliferation of germ cells contained in cysts, *i.e.* SpA_diff_ committed to spermatogenesis and type B spermatogonia, as well as (c) meiosis of committed spermatogonia to progress through spermatogenesis. On the contrary, we did not find any evidence that rFsh administration was effective in stimulating stem spermatogonia self-renewal. In fact, after 6 weeks of rFsh treatment, the density of anti-PCNA positive SpA_und_ decreased significantly compared to control fish and this reduction was so marked that only sparse stem spermatogonia self-renewal was observed in the peripheral region of the testis, which is usually the main spermatogonial proliferative region in teleosts, including meagre^41^. Therefore, the 6-week rFsh treatment appeared to stimulate differentiation of SpA_und_ and their progress towards spermatogenesis. This situation of induced spermatogenesis without stem spermatogonia self-renewal in the long term would be inviable as the available SpA_und_ would be exhausted and the present study, therefore, questions our understanding of the role of Fsh in stem spermatogonia self-renewal. The role of Gths in the reproductive process of fish is gradually being elucidated; however, the specific roles of Fsh and Lh have not yet been fully defined. It is generally accepted that Fsh acts on the early events of fish gametogenesis, whereas Lh acts on the final events of this process^42^. In vertebrates, it is widely recognised that Fsh exerts a proliferative role in the spermatogenic process^42–46^; however, the effect of Fsh alone in fish germ cell proliferation is still a matter of debate^37,47–49^. In several fish species, Fsh receptors have been detected both in Sertoli and Leydig cells^38,49–51^ and it has been proposed that Fsh triggers androgen production and release by Leydig cells^37^. Moreover, the increase of Fsh plasma levels observed at the onset of spermatogonial proliferation has been associated with mitosis of Sertoli cells^40^, whose increase in number is required to support the cyst enlargement associated with spermatogonial proliferation^52^. In the present study, an increase of seminiferous tubule diameter, associated with an increase of germinal epithelium height—and then with larger spermatocysts—was observed in rFsh-treated fish. In association with these histological changes, rFsh treated fish significantly increased 11-KT plasma levels compared to control fish (N.D. unpublished data).

In immature Japanese eel *Anguilla japonica*, treatments with 1.0 IU/g of rFsh induced an increase type B spermatogonia^36^, but no effect on proliferation of stem spermatogonia was reported. In this species, a similar effect was seen in vitro after 15 days of culture, where rFsh increased the percentage of late type B spermatogonia in a dose-dependent manner comparable to the effect of 11-KT^50^. In juvenile European sea bass *Dicentrarchus labrax* with quiescent testes, injections of single-chain rFsh increased the number of spermatogonia committed towards spermatogenesis, and cysts of spermatocytes and spermatids, thus confirming that Fsh is one of the early signals to proceed with spermatogenesis^37^. In yellowtail kingfish *Seriola lalandi* at the start of puberty during the spawning season, after rFsh was injected six times in 10-day intervals, spermatozoa were observed in the seminiferous tubules, whereas controls were still immature^53^. In Senegalese sole, rFsh induced spermatocyte and new spermatogenic tubule formation within 4 weeks^28^. In the present study, rFsh-treatment induced an advancement in spermatogenesis by reducing proliferation of undifferentiated spermatogonia and increasing proliferation of committed spermatogonia and their entry into meiosis.

Despite the widely documented effectiveness of Fsh in stimulating spermatogenesis in fishes -including steroidogenesis, spermatogonial proliferation and meiosis- neither the available literature nor the data obtained in the present study support a role of Fsh in stimulating spermatogonial self-renewal in pre-pubertal fish, which occurs throughout the spermatogenic cycle of both hermaphroditic^54^ and gonochoristic^33,34^ fishes and is likely necessary to support full testis growth and spermatogenesis over a prolonged spawning season. Studies on flathead grey mullet found a reduced production of sperm from males treated for 10 weeks with rFsh compared to males treated with both rFsh and rLh^31^ and a combined rFsh + rLh treatment for 11-weeks achieved GSIs of 5.35 ± 1.25 with production of fluent sperm in all treated males^32^. In European eel *A. anguilla*, GSI and testes development appeared to be dependent on both the duration and dose of rFsh treatment and was especially successful after the combined use of rFsh and rLh^30^. A 9-week rFsh treatment of 4 µg fish^−1^ rFsh (eel mean weight of 89.2 ± 5.4 g ≈ 44 µg kg^−1^ of rFsh) gave a GSI of 1 (compared to 0.2 at the start of the experiment), while a 12-week combined treatment of rFsh and rLh gave a GSI of 4.3 with good sperm production that was comparable to standardised human chorionic gonadotropin treatments. These studies did not examine stem spermatogonia self-renewal, but the increase in GSI and continued spermatogenesis may suggest that stem spermatogonia self-renewal was active when rLh was administered. However, this is speculative, and an alternative explanation would be that the maturation and hydration of the testes with seminal fluid increased testes size, and this agrees with the role that has been attributed to Lh. As generally accepted for Fsh, the rFsh treatments induced the early events of fish spermatogenesis through to production of spermatozoa, but rLh appears to be necessary to complete full testes growth. The mechanism underlying spermatogonial self-renewal is not yet elucidated; we have shown that spermatogonial self-renewal is low with the application of only rFsh and it remains to be determined how the additional application of rLh may function during testicular growth and maturation. This study suggests that future studies should examine the role of Fsh in stem spermatogonia renewal as the low levels of stem spermatogonia renewal implies that Fsh may not be involved in controlling renewal, which may be controlled by different pathways.

Germ cell apoptosis is an integral component of testicular function in fishes and the presence of apoptotic cells has been widely reported in all stages of the reproductive cycle, both in wild and cultured fishes^33,34,55–57^. In fish, as also shown in mammals, a role of apoptosis in the regulation of the ratio of germ cells to Sertoli cells, as well as in the prevention of aberrant germ cell development has been proposed^33,34,55–58^. In seasonal breeding mammals, increased rates of apoptosis occur during testicular regression, while little testicular apoptosis is observed during recrudescence or the breeding season^59–61^. Testosterone, the main androgen in mammals, is a cell survival factor for germ cells^60^ and withdrawal of Gths and T induces apoptosis in the testis^62,63^. In adult Atlantic bluefin tuna *Thunnus thynnus*^33,56,57^ and greater amberjack *Seriola dumerili*^34^ under captivity-induced chronic stress, the density of apoptotic male germ cells was significantly higher compared with wild breeders. Administration of GnRHa through sustained-release implants was effective in inducing a significant decrease of apoptotic germ cells in Atlantic bluefin tuna^56^, an effect likely associated to the increase of Gth and 11-KT plasma levels induced by the GnRHa treatment. In the present study, a high density of apoptotic germ cells was observed in untreated pre-pubertal meagre. A possible explanation of this finding is that spermatogonia that cannot proceed towards meiosis due to insufficient Gth and androgens die by apoptosis, and the rFsh treatment removed the apoptotic block allowing spermatogonia to proliferate and enter in meiosis. It is also interesting to observe that in the present study control fish had both renewal and apoptotic stem spermatogonia, indicating a turnover of germ cells that did not continue after rFsh treatment, where germ cells were stimulated to develop, and both renewal and apoptosis were lower. These observations highlight the dynamics of the testes in two different phases. In a stationary phase, the testes have a turnover of cells with higher levels in apoptosis and renewal, while in a phase of development stimulated by administration of rFsh, the testes focused almost entirely on germ cell development, while apoptosis and renewal were reduced.

Puberty in fish involves complex interactions among different endocrine axes/networks. Growth hormone has been reported to co-act with Gths in the regulation of the reproductive system^64–67^. Thyroid hormones are involved in sex differentiation, testicular development, growth and maturation^68^; in adult zebrafish *Danio rerio*, triiodothyronine (T3) expands the population of Sertoli cells and SpA_und_ with a mechanism involving insulin-like growth factor (Igf) signalling^69^. Moreover, leptin, a hormone secreted by adipocytes, plays a permissive role in puberty attainment when virgin fish attain a certain degree of somatic growth or a threshold of energy reserves^14^. The lack of the normal interplay between endocrine axes in sexually immature fish might represent a limiting factor for a full effectiveness of Gth-based treatments to induce precocious puberty. However, in the present study, no limit to the induction of puberty was observed and the rFsh treatment alone initiated puberty in all treated males.

## Conclusions

The present study demonstrated the effectiveness of rFsh produced in a CHO-S cell line in stimulating testis development and spermatogenesis in pre-pubertal meagre. A 6-week rFsh treatment proved to be highly efficient in removing the apoptotic block of spermatogenesis observed in juvenile meagre, allowing spermatogonial survival and progress towards meiosis. Further hormone co-treatments, along with rLh, may possibly help mimic the hormone axis cross talk necessary to induce full gonad maturation and sexual maturity in this species.

## Supplementary Information

Supplementary Information.

## Data Availability

Fsh α and β subunits amino acid sequences were deduced from the meagre pituitary mRNA sequences deposited in the European Nucleotide Archive (ENA) under the project accession number PRJEB57583. All the other data produced and/or analyzed during the current study are included in this article and in Supplementary Information file.
